# Perioperative Decision-Making for a Solitary Fibrous Tumor of the Pleura: Lessons from Two Cases and Current Evidence

**DOI:** 10.3390/diagnostics16071061

**Published:** 2026-04-01

**Authors:** Jung Hoon Yi

**Affiliations:** Department of Thoracic and Cardiovascular Surgery, Dong-A University Hospital, Dong-A University College of Medicine, Busan 49201, Republic of Korea; yjh051@dau.ac.kr; Tel.: +82-51-240-5195

**Keywords:** solitary fibrous tumor of the pleura, SFTP, perioperative management, VATS, risk stratification

## Abstract

**Background and Clinical Significance:** Solitary fibrous tumor of the pleura (SFTP), a rare mesenchymal neoplasm (<5% of pleural tumors), is often asymptomatic and incidentally detected. Preoperative differentiation between benign and malignant forms remains challenging, with 10–20% of lesions showing malignant behavior despite benign imaging. The role of conservative “watch-and-wait” versus early resection in small, asymptomatic cases is debated, prompting the need for structured perioperative decision-making based on imaging, pathology, and risk models. **Case Presentation:** We report two surgically resected SFTP cases. Case 1 involved an 8 cm giant pedunculated benign tumor managed by open thoracotomy (negative margins, mitotic count: 1.5–2.0/10 high-power fields, necrosis < 10%), with the patient remaining recurrence-free at 4 years postoperatively. Case 2 involved a 5.3 cm pedunculated benign tumor (initially 4.8 cm, which grew during a 2-year observation) excised via a minimally invasive technique (mitotic count: 0.5–1.5/10 high-power fields, necrosis < 10%), showing no recurrence over 6 months. In both cases, safe R0 resection was achieved through clinicoradiologic assessment and appropriate surgical technique selection. Both tumors were classified as benign and low risk by the England, Tapias, and modified Demicco models and are undergoing a tailored follow-up protocol. **Conclusions:** These two cases illustrate the feasibility of en bloc R0 resection in operable SFTP, despite interval growth observed in Case 2. Combined with literature evidence, early en bloc R0 resection appears to be preferable to prolonged observation for operable SFTP, balancing the risks of unpredictable growth, malignant transformation, and recurrence—even in histologically benign tumors (up to 6.3%)—against potential surgical morbidity. Tailored preoperative planning appears beneficial, complemented by pathology-based risk models for postoperative stratification and risk-adapted surveillance.

## 1. Introduction

Solitary fibrous tumor of the pleura (SFTP) is a rare mesenchymal neoplasm accounting for <5% of all pleural tumors [1]. Advances in immunohistochemistry (CD34, STAT6) and molecular profiling (NAB2-STAT6 fusion) have established it as a distinct entity, separate from mesothelioma. While most cases (80–90%) follow a benign course, 10–20% of lesions exhibit malignant behavior, resulting in local recurrence, distant metastasis, or death [2,3].

Patients are often asymptomatic (>50%), and tumors are incidentally detected on chest imaging [4]. Growth can cause compressive symptoms (cough and dyspnea) or rare paraneoplastic syndromes (Doege–Potter and Pierre–Marie–Bamberger) [4,5]. Preoperative benign–malignant distinction is difficult: needle biopsy yields low diagnostic accuracy and carries a risk of tumor seeding [5,6]. Thus, en bloc R0 resection is widely regarded as both the definitive diagnostic and therapeutic approach [1,7].

Prior studies recommended tailoring surgical approaches based on tumor characteristics. For small tumors that are presumed benign, minimally invasive techniques, including video-assisted thoracoscopic surgery (VATS) are favored, while giant or sessile lesions typically warrant open approaches to minimize rupture risk [8,9,10]. Even after complete resection, late recurrences (>10 years) have been reported, prompting the development of various postoperative prognostic models that use resected specimens [5,10].

Debate persists regarding “watch-and-wait” versus early resection for small, asymptomatic, radiologically benign-appearing SFTP. Despite systematic reviews that favor surgery, direct comparisons of early versus delayed resection remain limited. Our report presents two cases (one giant, incidentally discovered tumor and one case with observed growth over time) to contextualize perioperative decision-making—from imaging assessment and timing to approach selection and risk stratification—within current evidence.

## 2. Case Report

### 2.1. Case 1 (Open Thoracotomy Case)

A 57-year-old male was referred to our thoracic surgery department in January 2022 after an abnormal radiopacity was identified on a routine screening chest radiograph. The patient was completely asymptomatic and had no significant past medical or family history. Physical examination and preoperative evaluations, including laboratory tests, electrocardiography, and pulmonary function tests (PFTs), were all within normal limits.

A chest computed tomography (CT) scan revealed an approximately 8 cm, well-circumscribed mass in the left hemithorax. The tumor formed an obtuse angle with the adjacent pleural surface and exhibited mildly heterogeneous contrast enhancement internally, strongly suggesting a giant SFTP. A limited posterolateral thoracotomy was planned to minimize the risk of capsular rupture associated with the massive size of the tumor and to ensure adequate surgical exposure.

**Figure 1:**
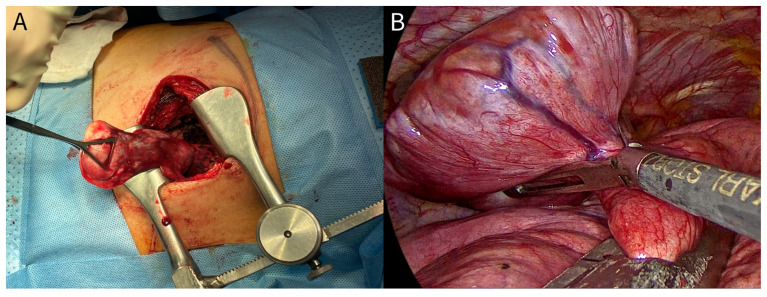
Intraoperative views of the solitary fibrous tumors of the pleura. (**A**) Case 1: the intraoperative view via a limited posterolateral thoracotomy revealing an elongated, well-circumscribed, lobulated mass. (**B**) Case 2: the thoracoscopic view demonstrating an oval-shaped, pedunculated, solid mass attached to the underlying lung parenchyma. En bloc resection without capsular rupture was successfully performed for both tumors.

During surgical exploration, the tumor was identified as a large, oval-shaped mass connected to the visceral pleura of the left lower lobe via a pedicle (Figure 1A). There were no signs of direct invasion or dense adhesions to the chest wall, mediastinum, or diaphragm. An en bloc resection of the tumor, including a wedge resection of the underlying lung parenchyma at the pedicle base, was successfully performed without any intraoperative tumor spillage.

**Figure 2:**
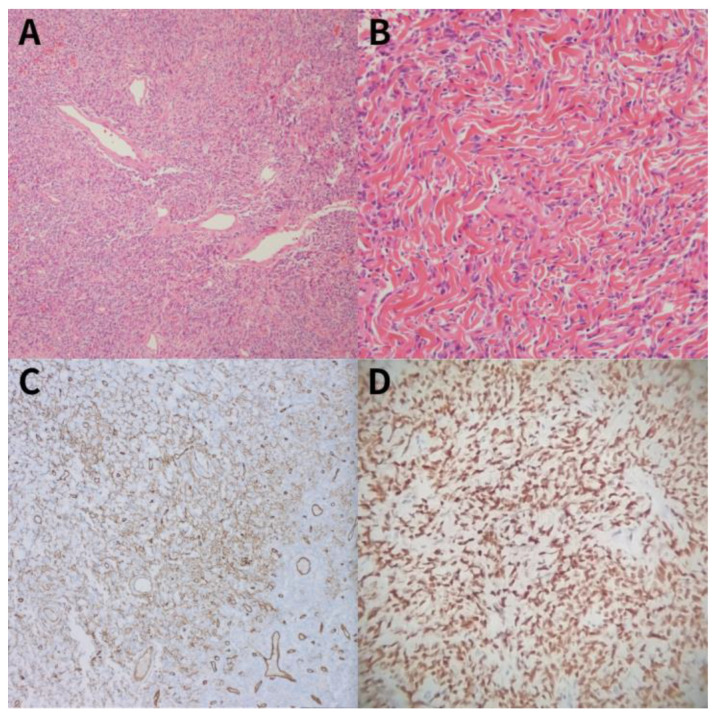
Histopathological features of the resected solitary fibrous tumor. (**A**) The tumor shows a characteristic “patternless pattern” composed of spindle cells randomly arranged around branching blood vessels (H&E stain, ×100). (**B**) Areas of stromal hyalinization with keloid-like collagen deposition are observed (H&E stain, ×400). (**C**) Immunohistochemical staining shows strong and diffuse positivity for CD34 (×200). (**D**) The tumor cells exhibit strong nuclear reactivity for STAT6 (×200), confirming the diagnosis.

Macroscopic examination of the resected specimen revealed a well-circumscribed, lobulated mass with prominent hemorrhage on the cut surface. Histopathological examination demonstrated a “patternless pattern” of spindle cells arranged around blood vessels (Figure 2A), accompanied by stromal hyalinization resembling keloid-like deposition (Figure 2B). Immunohistochemical staining showed strong positivity for CD34 and STAT6 (Figure 2C,D). Furthermore, all resection margins were confirmed to be negative for tumor cells. Subsequent histological evaluation for prognostic profiling revealed a mitotic index of 1.5–2.0 per 10 high-power fields (HPFs) with minimal necrosis (<10%). Consequently, the tumor was classified as a low-risk SFTP according to the modified Demicco model.

The patient had an uneventful postoperative recovery, was discharged without complications, and remains free of recurrence at the 4-year follow-up.

### 2.2. Case 2 (VATS Case)

**Figure 3:**
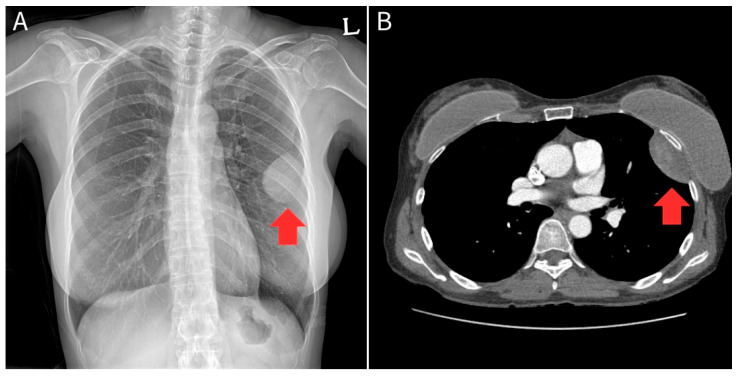
Preoperative radiological findings of the patient in Case 2. (**A**) The posteroanterior chest radiograph reveals a well-defined, rounded radiopacity in the left middle lung zone. (**B**) The chest computed tomography scan demonstrates a 5.3 cm, well-circumscribed, solid mass in the left hemithorax. The tumor forms an obtuse angle with the adjacent mediastinal pleura near the lingular segment, indicating its pleural origin. The mass appears homogeneous without evidence of calcification, necrosis, or invasion into surrounding structures.

A 53-year-old female was referred to our department in July 2025 due to an enlarging pleural mass. Initially, a 4.8 cm mass in the left hemithorax was detected incidentally during a health screening examination in December 2022. She had been followed for >2 years at the Department of Pulmonology with a suspected diagnosis of SFTP. Although she remained asymptomatic, a follow-up chest CT scan in July 2025 revealed that the tumor had increased in size to 5.3 cm (Figure 3). PFTs showed that while forced expiratory volume in 1 s (FEV1) and forced vital capacity (FVC) values were within normal limits, the FEV1/FVC ratio was slightly reduced to 69%.

Preoperative evaluation revealed no evidence of intrathoracic lymphadenopathy or distant metastasis. After confirming the absence of surgical contraindications, tumor resection via VATS was performed in September 2025. Under thoracoscopic visualization, the tumor presented as an oval-shaped solid mass attached to the lingular segment of the left upper lobe via a pedicle, without adhesions to the chest wall or mediastinum (Figure 1B). Consequently, an en bloc resection of the tumor along with the underlying lung parenchyma was performed.

The histopathological examination revealed a tumor measuring 5.3 × 4.8 cm, with histological features consistent with a typical benign SFTP. The tumor cells exhibited immunoreactivity for CD34 and STAT6. Additionally, the surgical resection margins were microscopically clear of tumor cells. Further pathological assessment for risk stratification demonstrated a mitotic count of 0.5–1.5 per 10 HPFs, and the extent of tumor necrosis was <10%. Based on the modified Demicco model, the lesion was stratified as low risk.

The patient was discharged without complications on postoperative day 2, showing no evidence of recurrence at their most recent 6-month follow-up visit.

## 3. Discussion

### 3.1. Imaging Characteristics and Predictors

The first critical step in managing SFTP is accurate radiological evaluation [11]. CT plays the most important role in identifying the tumor’s anatomical location, size, and relationship with adjacent organs. Typical benign SFTP appears on chest CT as a single, well-defined, smooth mass forming an obtuse angle suggestive of pleural origin [12]. Small tumors show homogeneous enhancement, while larger tumors often exhibit heterogeneous enhancement due to intratumoral necrosis, cystic degeneration, or myxoid stroma [13].

Although imaging findings alone cannot perfectly differentiate benign from malignant SFTP, certain features strongly suggest malignancy or aggressive clinical behavior (Table 1) [14]. First, giant tumors > 10 cm show a high correlation with malignant pathology. Second, ill-defined margins and direct invasion into adjacent structures, such as the chest wall, mediastinum, or lung parenchyma, also indicate malignant behavior [15]. Third, accompanying pleural effusion, multiple nodules, or extensive internal necrosis should raise suspicion of malignant transformation [14]. In case 2, the patient initially presented with a 4.8 cm mass with clear margins, showing typical benign imaging features; however, the size increased over 2 years. Hence, even benign-appearing imaging findings may harbor ongoing biological activity, highlighting the clinical limitations of relying solely on imaging to predict long-term tumor behavior.

### 3.2. Limitations of the ‘Watch-And-Wait’ Strategy and Rationale for Definitive Surgical Resection

According to recent systematic reviews and large multicenter studies, conservative observation is generally not recommended as a primary strategy for SFTP [5,10,15,16,17,18,19,20]. Although SFTPs typically exhibit slow growth, they can unpredictably enlarge over time, potentially causing compression of adjacent organs, increasing surgical difficulty, and raising the risk of malignant transformation [5,16,17]. Furthermore, tumor recurrence (overall 4.9–16.7%) is not exclusively confined to malignant cases (Table 2); even histologically benign SFTPs can recur (benign: 0–6.3%; malignant: 22–33%), underscoring the unpredictable clinical course that should be considered when selecting an observational approach. Most importantly, modern risk stratification models—which are essential for predicting recurrence and guiding long-term surveillance—rely fundamentally on detailed pathological criteria, such as mitotic count and tumor necrosis [16,21,22]. These critical parameters can only be accurately assessed following surgical resection. Nevertheless, recognizing the exceptionally indolent clinical course of many SFTPs, a ‘watch-and-wait’ approach may still be a reasonable alternative for a highly selected group of frail or high-risk surgical candidates.

### 3.3. Surgical Strategies for Safe R0 Resection: VATS Versus Thoracotomy

The primary oncologic goal while treating SFTPs is achieving complete resection (R0 resection) without capsular rupture. The choice of surgical approach (VATS or thoracotomy) should be individualized based on tumor size, location, attachment pattern (pedunculated vs. sessile), and the surgeon’s expertise [10,20].

Recent retrospective studies and meta-analyses have indicated that VATS provides equivalent oncologic outcomes (overall survival and disease-free survival) to thoracotomy for pedunculated tumors < 5–8 cm, with clear advantages in reduced postoperative pain, shorter hospital stay, and better cosmesis [6,18]. In case 2, the increase from 4.8 cm to 5.3 cm did not preclude a thoracoscopic approach, as the tumor remained pedunculated, with clear dissection planes and an easily controllable vascular pedicle. From a technical standpoint, VATS resection was still straightforward, and en bloc extraction was feasible. However, retrieving the intact specimen from the rigid thoracic cavity becomes increasingly challenging as tumors enlarge. Extending the utility incision or retracting the ribs to extract a larger mass fundamentally negates the minimally invasive benefits of VATS.

For giant tumors ≥ 8 cm (such as in case 1), lesions with a broad pleural base attachment, or those with imaging suggesting malignancy, open surgery such as thoracotomy or median sternotomy remains the standard [18,20]. Attempting VATS on giant tumors increases the risk of capsular rupture or tumor spillage during extraction, which is the most fatal risk factor for local recurrence. Thus, the overriding principle must be “safe” R0 resection regardless of approach.

### 3.4. Pathologic Features and Comparison of Risk Stratification Systems

Precise pathologic analysis of the resected tumor is essential for prognosis prediction and follow-up planning. Histologically, SFTP diagnosis is confirmed by characteristic ovoid and spindle cell distribution, along with immunohistochemical positivity for CD34, Bcl-2, and the most specific marker, nuclear STAT6 expression [8]. Molecularly, nearly all SFTP cases feature NAB2-STAT6 gene fusion on chromosome 12q13 [8,14].

Over the past 20 years, various scoring systems have been developed to stratify long-term prognosis and recurrence risk in SFTP patients (Table 3).

**Table 3:** Comparison of Major Risk Stratification Systems for Solitary Fibrous Tumor.

	England [21]	Tapias [16]	Modified Demicco [22]	Case 1	Case 2
**Histologic criteria**					
Mitotic count (/10 high-power fields)	>4	≥4	0 = 0 1–3 = 1 ≥4 = 2	1.5–2.0 = 1	0.5–1.5 = 1
Hypercellularity	Yes	Yes		No	No
Pleomorphism	Yes			No	No
Necrosis (or hemorrhage)	Yes	Yes	<10% = 0 ≥10% = 1	<10% = 0	<10% = 0
**Anatomic criteria**					
Pleural origin		Parietal		Visceral	Visceral
Morphology		Sessile		Pedunculated	Pedunculated
Size (cm)		≥10	<5 = 0 5–10 = 1 10–15 = 2 ≥15 = 3	8 cm = 1	5.3 cm = 1 (initially 4.8 cm = 0)
**Patient criteria**					
Age			≥55 = 1	57 = 1	53 = 0
**Classification**	Malignant if ≥1 criteria	High risk for recurrence if ≥3 criteria	Metastatic risk Low = 0–3 Intermediate = 4–5 High = 6–7	**England**: benign **Tapias**: low risk **Demicco**: 3 = low risk	**England**: benign **Tapias**: low risk **Demicco**: 2 = low risk

The most classical system is the England criteria [21], which relies solely on pathologic factors and classifies a lesion as malignant if ≥1 of the following is met: (1) hypercellularity, (2) pleomorphism, (3) mitotic count > 4 mitoses per 10 HPFs, and (4) presence of necrosis or hemorrhage. However, it overlooks key clinical factors such as tumor size and resection completeness.

To address this, the Tapias scoring system was proposed [16]. Tapias et al. devised a combined clinical-pathologic score evaluating (1) tumor size (≥10 cm vs. <10 cm), (2) pleural origin (visceral vs. parietal), (3) morphology (sessile vs. pedunculated), (4) mitotic count ≥ 4 mitoses per 10 HPFs, (5) hypercellularity, and (6) presence of necrosis or hemorrhage for refined recurrence risk assessment. This score ranges from 0 to 6 (low risk < 3, high risk ≥ 3).

The most recently established modified Demicco risk stratification system sums the patient’s age (≥55 years), tumor size (5 cm, 10 cm, and 15 cm thresholds), mitosis, and necrosis percentage to classify tumors as low-, intermediate-, or high-risk [22]. This risk prediction model was not intended only for SFTPs and could also be used for extrathoracic solitary fibrous tumors (SFTs) [18]. Comparative studies confirmed that the modified Demicco model outperforms the England criteria in predicting distant metastasis and disease-specific survival [14,15]. A 2025 20-year retrospective study confirmed that the modified Demicco score offers the highest accuracy for recurrence prediction in SFTP, with recurrence sharply increasing mortality [19].

Both cases presented in this report were classified as benign based on the England criteria and as low risk by both the Tapias and modified Demicco systems (Table 3). In the second case, an interval before surgery allowed the tumor to enlarge from 4.8 cm to 5.3 cm, increasing the size score by 1 point in the modified Demicco system. Although the tumor maintained its low-risk grade due to favorable scores in the remaining criteria, this 1-point addition had the potential to upstage the classification to intermediate or high risk otherwise.

Diebold et al. emphasized the importance of the cell proliferation marker MIB-1 (Ki-67) labeling index for assessing malignancy in a multicenter study [23]. With reports of recurrence in histologically benign-appearing cases, MIB-1 indices ≥5–10% indicate a high risk of malignant-like behavior despite benign histology [14,15]. Another recent study has evaluated the integration of preoperative Positron Emission Tomography–CT (PET/CT) maximum standardized uptake (SUVmax; with a cutoff value of 4.50) alongside the modified Demicco model in a cohort of 69 patients with pleuropulmonary solitary fibrous tumors [24]. Incorporating this SUVmax threshold successfully categorized all 6 relapsed patients as high risk, including 4 who had been classified as intermediate risk under the modified Demicco system. Although the MIB-1 labeling index and PET/CT imaging were not utilized in the present cases, they may serve as auxiliary tools for assessing the malignant potential and recurrence risk of these tumors.

### 3.5. Risk-Stratified Surveillance Strategies

Recent studies comparing risk stratification models have demonstrated the superior utility of the modified Demicco system in predicting recurrence [19,25]. Furthermore, the WHO Classification of Tumors of Soft Tissue and Bone (5th ed., Vol. 3, 2020) and Thoracic Tumors (5th ed., Vol. 5, 2021) [26,27] abandoned classical terminology such as “typical (benign)” or “malignant” for SFT, instead recommending the modified Demicco risk assessment model as a superior tool for determining prognosis [28,29].

SFTP is explicitly included as a target disease in the NCCN and ESMO soft tissue sarcoma guidelines [30,31]. Given the rarity of such lesions, which precludes large-scale studies or randomized controlled trials, reference to follow-up recommendations within these guidelines can be clinically helpful. Although neither guideline addresses SFT as a separate entity, both advocate risk-adapted surveillance intensity across multiple soft tissue sarcomas. Decker et al. emphasized the superiority of the modified Demicco system through a multicenter international study and proposed a risk-stratified follow-up protocol [25].

We adopted elements from both international guidelines in our follow-up protocol. Low-risk patients undergo surveillance imaging every 6 months for the first year, annually for the subsequent 4 years, and biennially thereafter. Intermediate- and high-risk patients receive imaging every 3 months in the first year, twice yearly up to 5 years, and annually thereafter. Considering the well-documented risk of delayed relapse, with the literature reporting recurrences 10–20 years after initial resection, continued long-term surveillance remains highly advisable [29].

## 4. Conclusions

**Figure 4:**
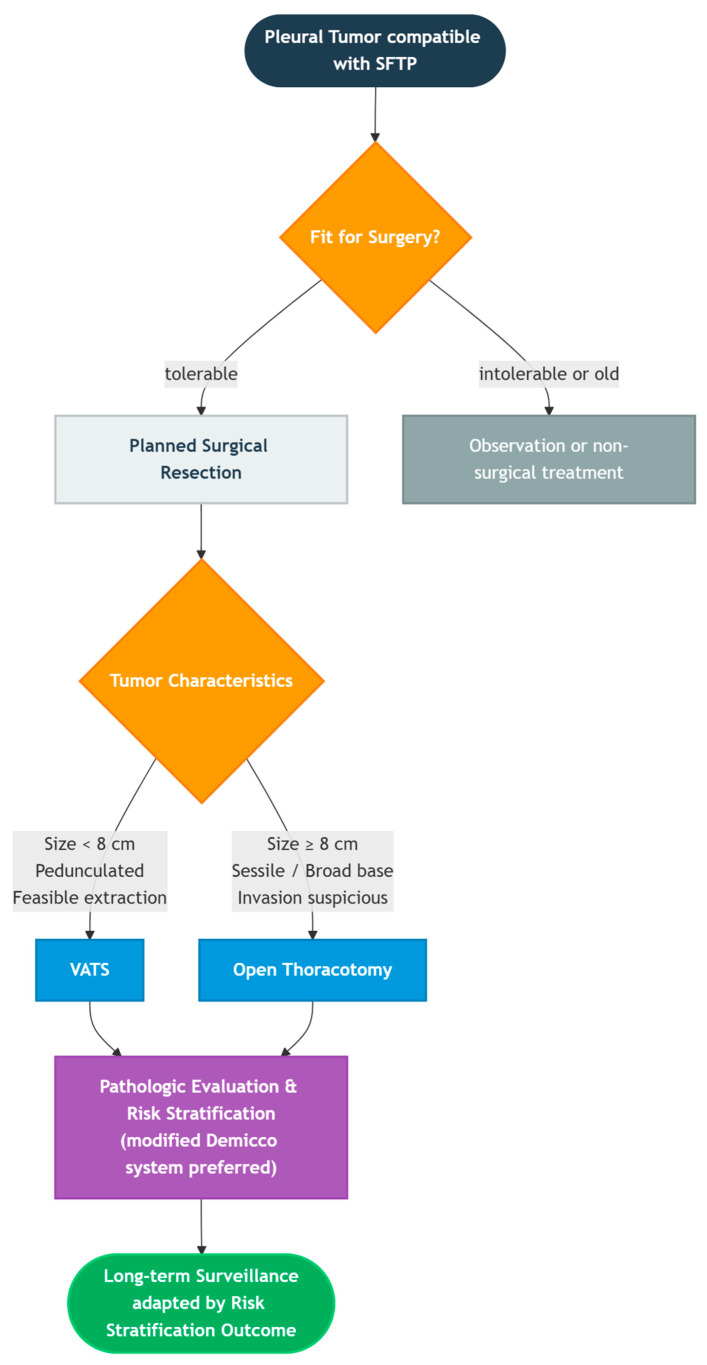
Practical perioperative decision-making algorithm summarizing our approach and current evidence for the management of solitary fibrous tumors of the pleura (SFTPs).

SFTP management may benefit from a structured perioperative approach based on our institutional experience and current evidence (Figure 4). First, when imaging suggests a pleural-based solitary fibrous tumor, multidisciplinary discussion can help weigh early en bloc R0 resection against short-term observation in selected frail patients. Second, the choice between minimally invasive and open surgery is best individualized according to tumor size, morphology, location, and expected ease of safe specimen retrieval. Third, the postoperative integration of pathology with contemporary risk models, such as the modified Demicco and Tapias systems, can support prognostic stratification. Finally, follow-up intensity should be adapted to the estimated risk, with long-term surveillance considered prudent given the potential for late recurrence.

## Figures and Tables

**Table 1:** Imaging features favoring benign vs. malignant solitary fibrous tumor of the pleura.

Feature	Benign Favoring	Malignant Favoring
Tumor Size	≤10 cm	>10 cm
Shape	ovoid/round, smooth, or gently lobulated mass	irregularly lobulated, coarse and asymmetric contour
Margin	well-defined, smooth	ill-defined or spiculated
Angle with pleura	obtuse angle	acute angle, appearing to insinuate into the lung parenchyma
Internal Structure	homogeneous soft-tissue attenuation	heterogeneous attenuation with extensive low-attenuation areas representing necrosis, cystic change, or hemorrhage
Attachment	broad-based or pedunculated	sessile
Associated Findings	preserved fat planes	pleural effusion or multiple nodules

**Table 2:** Summary of key outcomes in the recent literature. In Lococo’s study, the resection was complete in 46 cases (92%). NR, not reported; yr, year; DFS, disease-free survival.

Study (Year)	Type	Patient (Benign/Malignant)	Median Follow-Up (yr)	Overall Recurrence (%)	Recurrence (Benign) (%)	Recurrence (Malignant) (%)	Median Time to Recurrence (yr)	Overall Survival (%)	Survival (Benign)	Survival (Malignant)
Mercer et al. (2020) [5]	Systematic review and meta-analysis (27 studies)	1299 (905/394)	NR	7.9–13.1	2.8	NR	NR 5 yr DFS: 71–87.2%	5 yr: 83–92.4%	NR	NR
Liu et al. (2021) [6]	Systematic review and meta-analysis (23 studies)	1262 (NR/NR)	NR	9	3	22	NR	NR	NR	NR
Bellini et al. (2019) [15]	Retrospective	107 (79/28)	7	11.2	6.3	25	9.1 10 yr DFS: 81%	NR disease-related death: 7.5%	NR	NR
Tan et al. (2018) [17]	Retrospective	82 (70/12)	4.7	4.9	0	33	NR	NR	NR	76%
Lococo et al. (2012) [3]	Retrospective	50 (0/50)	4.4	30	-	30	2.8	5 yr: 81.1, 10 yr: 66.9	-	5 yr: 81.1, 10 yr: 66.9
Tapias et al. (2013) [16]	Retrospective	59 (NR/NR)	8.8	14	NR	NR	6 5 yr DFS: 87.2%, 10 yr DFS: 72.1%	5 yr: 92.4, 10 yr: 83.4	NR	NR
Gagnepain et al. (2025) [19]	Retrospective	96 (NR/NR)	6	16.7	NR	NR	5 yr DFS: 88.4%	5 yr: 93.9% disease-related death: 6.3%	NR	NR

## Data Availability

The data presented in this study are available on request from the corresponding author. The data are not publicly available due to privacy restrictions.

## Abbreviations

The following abbreviations are used in this manuscript:

SFTP	Solitary fibrous tumor of the pleura
SFT	Solitary fibrous tumor
HPF	High-power field
NR	Not reported
FEV1	Forced expiratory volume in 1 s
FVC	Forced vital capacity
VATS	Video-assisted thoracoscopic surgery
CT	Computed tomography
DFS	Disease-free survival
FDG	Fluorodeoxyglucose
PET/CT	Positron emission tomography–computed tomography
SUVmax	Maximum standardized uptake
